# Sub-1.4eV bandgap inorganic perovskite solar cells with long-term stability

**DOI:** 10.1038/s41467-019-13908-6

**Published:** 2020-01-09

**Authors:** Mingyu Hu, Min Chen, Peijun Guo, Hua Zhou, Junjing Deng, Yudong Yao, Yi Jiang, Jue Gong, Zhenghong Dai, Yunxuan Zhou, Feng Qian, Xiaoyu Chong, Jing Feng, Richard D. Schaller, Kai Zhu, Nitin P. Padture, Yuanyuan Zhou

**Affiliations:** 1 Faculty of Material Science and Engineering, Kunming University of Science and Technology, Kunming, Yunan 650093 China; 2 School of Engineering, Brown University, Providence, RI 02912 USA; 3 Center for Nanoscale Materials, Argonne National Laboratory, Lemont, IL 60439 USA; 4 Advanced Photon Sources, Argonne National Laboratory, Lemont, IL 60439 USA; 5 Department of Chemistry, Northwestern University, Evanston, IL 60208 USA; 6 Chemical and Nanoscience Center, National Renewable Energy Laboratory, Golden, CO 80401 USA

**Keywords:** Chemistry, Energy science and technology, Solar energy, Materials science, Physics

## Abstract

State-of-the-art halide perovskite solar cells have bandgaps larger than 1.45 eV, which restricts their potential for realizing the Shockley-Queisser limit. Previous search for low-bandgap (1.2 to 1.4 eV) halide perovskites has resulted in several candidates, but all are hybrid organic-inorganic compositions, raising potential concern regarding device stability. Here we show the promise of an inorganic low-bandgap (1.38 eV) CsPb_0.6_Sn_0.4_I_3_ perovskite stabilized via interface functionalization. Device efficiency up to 13.37% is demonstrated. The device shows high operational stability under one-sun-intensity illumination, with *T*_80_ and *T*_70_ lifetimes of 653 h and 1045 h, respectively (*T*_80_ and *T*_70_ represent efficiency decays to 80% and 70% of the initial value, respectively), and long-term shelf stability under nitrogen atmosphere. Controlled exposure of the device to ambient atmosphere during a long-term (1000 h) test does not degrade the efficiency. These findings point to a promising direction for achieving low-bandgap perovskite solar cells with high stability.

## Abstract (web summary)

Current research focus on the perovskites solar cells (PSCs) is mainly limited to the lead-based ones with bandgaps above 1.5 eV. Here Hu et al. report efficient and stable inorganic tin-containing PSCs, opening doors to exploring abundant perovskite materials with bandgaps lower than 1.4 eV.

## Introduction

Perovskite solar cells (PSCs), which employ halide perovskites with the AMX_3_-structure as light absorbers, have emerged as a potentially disruptive technology in the field of thin-film photovoltaics^1–6^. The power conversion efficiency (PCE) of PSCs has increased rapidly to a certified record of 25.2%^2^ in the past decade, since the first PSC report by Miyasaka and coworkers in 2009 (ref. ^7^). While halide perovskites are extremely versatile in composition, the perovskite absorber materials in the state-of-the-art PSCs are generally based on CH_3_NH_3_PbI_3_ (MAPbI_3_) and HC(NH_2_)_2_PbI_3_ (FAPbI_3_). Microstructural tailoring of MAPbI_3_- and FAPbI_3_-based perovskite thin films has led to the realization of nearly full potential of MAPbI_3_- and FAPbI_3_-based perovskites as light absorbers, which is a major driving force pushing the technological progress of PSCs^1,8^. Nevertheless, the state-of-the-art perovskites still have bandgaps larger than 1.45 eV (that of pure FAPbI_3_)^9^. This limits the highest attainable PCE in single-junction devices according to Shockley–Queisser (S–Q) limit, which predicts an ideal bandgap of 1.2–1.4 eV^10^. Thus, there is an unambiguous need to develop perovskites materials with lower (or more ideal) bandgaps. Note that for the ease of description and comparison, we treat all perovskite materials that exhibit bandgaps lower than pure FAPbI_3_ (1.45 eV) in the category of low-bandgap perovskites.

There have been several reports in the literature in this important direction of perovskites research, demonstrating the feasibility of compositions such as (FAPbI_3_)_0.7_(CsSnI_3_)_0.3_ (ref. ^11^), (FASnI_3_)_0.6_(MAPbI_3_)_0.4_ (refs. ^12,13^), (FASnI_3_)_0.6_(MAPbI_3_)_0.34_(MAPbBr_3_)_0.06_ (ref. ^14^), FAPb_0.5_Sn_0.5_I_3_ (ref. ^15^), and MAPb_0.5_Sn_0.5_I_3_ (ref. ^16^) for PSCs. Low-bandgap PSCs with PCE as high as 20.5% and 21.1% have been recently demonstrated by Tong et al.^17^, using an additive-mediated crystallization process, and by Lin et al.^18^, using a so-called ‘comproportionation’ approach. It is clear that continued improvements in the processing and microstructural engineering of low-bandgap perovskite thin films will further boost the PCE, which may eventually surpass the PCEs of the state-of-the-art PSCs. However, all the reported high-performance low-bandgap perovskites invariably contain a significant portion of organic component (MA^+^ or FA^+^) occupying the A-sites in the AMX_3_ perovskite structure, where M is Pb^2+^ or/and Sn^2+^ and X is a halide anion. The A-site organic cations, especially MA^+^, are often associated with high hygroscopicity and low thermal stability^19,20^, raising significant concerns regarding their long-term stability. To date, the best reported stability in low-bandgap PSCs shows a modest *T*_80_ device-operation lifetime of 222 h^17^.

In this work, we demonstrate the use of an inorganic perovskite composition of CsPb_0.6_Sn_0.4_I_3_ with a low bandgap of 1.38 eV for PSCs. The inorganic nature renders this perovskite inherently more stable than its hybrid organic–inorganic counterparts. Importantly, it is shown that a rational strategy of interface functionalization further stabilizes the CsPb_0.6_Sn_0.4_I_3_ perovskite thin films and passivates the defects, making CsPb_0.6_Sn_0.4_I_3_ perovskite a promising candidate for use in low-bandgap PSCs. High-PCE PSC devices are achieved with promising long-term operational and shelf stabilities.

## Results

### Synthesis and properties of CsPb_0.6_Sn_0.4_I_3_ perovskites

**Fig. 1:**
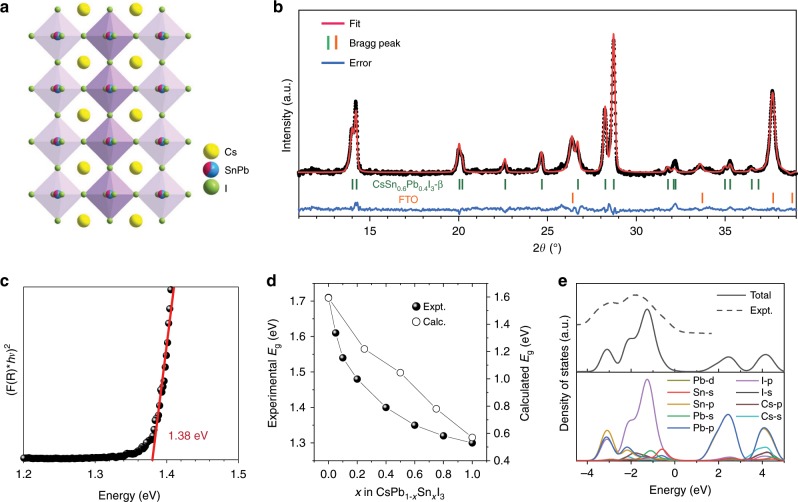
Synthesis and optical properties of CsPb_0.6_Sn_0.4_I_3_ perovskites. **a** Schematic crystal structure of CsPb_1−*x*_Sn_*x*_I_3_. **b** XRD pattern of CsPb_0.6_Sn_0.4_I_3_ perovskite thin film and its Rietveld refinement. **c** Tauc plot of CsPb_0.6_Sn_0.4_I_3_ perovskite thin film. **d** Variation of experimental (solid circle) and calculated (open circle) bandgaps as a function of *x* in CsPb_1−*x*_Sn_*x*_I_3_. **e** DOS of a typical CsPb_1−*x*_Sn_*x*_I_3_ (*x* = 0.5) perovskite calculated using DFT. Dashed curve is experimental UPS spectrum.

The CsPb_0.6_Sn_0.4_I_3_ perovskite is a solid-solution alloy phase (shown schematically in Fig. 1a) of CsPbI_3_ and CsSnI_3_, which is synthesized using the ‘one-step’ solution-processing approach (see details in Methods). The X-ray diffraction (XRD) pattern of as-synthesized CsPb_0.6_Sn_0.4_I_3_ thin films is shown in Fig. 1b. Rietveld refinement suggests a tetragonal β phase, with space group *P4/mbm* (lattice parameters: *a* = *b* = 8.78 Å; *c* = 6.31 Å), at room temperature (RT). In Supplementary Fig. 1, the UV-vis absorption spectrum of a CsPb_0.6_Sn_0.4_I_3_ perovskite thin film shows full-range of light absorption from the UV to near-infrared regions. The Tauc plot in Fig. 1c indicates that CsPb_0.6_Sn_0.4_I_3_ perovskite exhibits a bandgap of 1.38 eV. For comparison, the bandgap of the CsPbI_3_ perovskite is 1.73 eV^21–23^. The variation of experimental bandgaps as a function of *x* in CsPb_1−*x*_Sn_*x*_I_3_ has been further studied, which shows a decreasing bandgap with increasing *x*, as shown in Fig. 1d. This is in good agreement with the results from density functional theory (DFT) calculations. However, the bandgap ‘bowing’ effect^24^, which is typically observed in hybrid Pb–Sn alloy perovskites, is not observed in our inorganic CsPb_1−*x*_Sn_*x*_I_3_, which could be related to the absence of organic cations. The projected density of states (DOS) profile of CsPb_1−*x*_Sn_*x*_I_3_ alloy (*x* = 0.5) is presented in Fig. 1e, together with the experimental ultraviolet photoelectron spectrum (UPS), which captures the overall valence-band states. Here, the substitution of Pb^2+^ by Sn^2+^ in the AMX_3_ structure narrows the bandgap by pushing the valence band maximum (VBM) up, as Sn^2+^ cations exhibit higher-energy occupied *s*^2^ orbitals that couple with I *p* orbitals^9^. Interestingly, the experimentally measured change in the bandgap (*E*_g_) with *x* does not strictly follow the Vegard’s law (linear relationship) in CsPb_1−*x*_Sn_*x*_I_3_. Instead, an empirical biexponential function is needed to fit the descending trend, leading to an initially steep decrease in the bandgap when only small amount of Sn^2+^ is incorporated. This offers the opportunity for achieving the desired low bandgaps in CsPb_1−*x*_Sn_*x*_I_3_ perovskites with a relatively small value of *x* = 0.4. Note that a relatively low content of Sn^2+^ makes the manipulation of material properties much easier because the concentration of undesirable Sn-related defects, which are dominant in Sn-containing halide perovskites^25^, is expected to be lower. In this context, preliminary device effort (Supplementary Fig. 2) suggests that it is more challenging to make higher-Sn-content CsPb_1−*x*_Sn_*x*_I_3_-based PSCs even if they offer the prospect of lowering the bandgap. Thus, the CsPb_0.6_Sn_0.4_I_3_ composition appears optimum as it combines the merits of good structural stability and the stability of the Sn^2+^ cations.

The low-bandgap CsPb_0.6_Sn_0.4_I_3_ perovskite could be advantageous over their hybrid organic–inorganic counterparts in terms of easier solution-processing and higher intrinsic structural stability. Supplementary Fig. 3 shows that the one-step spin-coating method can be used without any antisolvent treatment to deposit uniform CsPb_0.6_Sn_0.4_I_3_ perovskite thin films. In contrast, their hybrid counterparts show poor film coverage in the absence of additional antisolvent treatment. CsPb_0.6_Sn_0.4_I_3_ perovskite exhibits improved phase stability compared to the widely studied CsPbI_3_ perovskite. As seen in Supplementary Fig. 4, the CsPb_0.6_Sn_0.4_I_3_ perovskite thin film remains black (indication of the perovskite phase retention) upon heating at 80 °C for up to 18 min in a nitrogen-filled glovebox, whereas the CsPbI_3_ perovskite thin film turns yellow quickly. This appears to be related to the Goldschmidt tolerance factor, which determines the stability of the perovskite structure: $t = \frac{{r_{\mathrm{A}} + r_{\mathrm{X}}}}{{\sqrt 2 \left( {r_{\mathrm{M}} + r_{\mathrm{X}}} \right)}}$^26^, where *r*_A_, *r*_M_, and *r*_X_ are radii of A, M, and X cations, respectively, in the AMX_3_ perovskite structure. Partial substitution of Pb with smaller Sn cation may result in a more ideal *t* value, which resembles the case of CsSn_0.5_Ge_0.5_I_3_ perovskite reported by us earlier^27^. We have further calculated the formation energy of CsPb_1−*x*_Sn_*x*_I_3_ (Supplementary Fig. 5), which shows a thermodynamically more stable crystal structure with Sn^2+^ substitution. The phase stability could also be related to its unique crystal symmetry (tetragonal β phase)^28^. However, CsPb_0.6_Sn_0.4_I_3_ perovskite is still not sufficiently stable under environmental conditions. When a neat CsPb_0.6_Sn_0.4_I_3_ perovskite thin film is exposed to ambient condition in air with high relative humidity (RT; 80% RH), it turns yellow quickly (Supplementary Fig. 6). To mitigate this environmental degradation issue, an interface-functionalization approach is employed, as described below.

### Interface-functionalization of CsPb_0.6_Sn_0.4_I_3_ perovskites

**Fig. 2:**
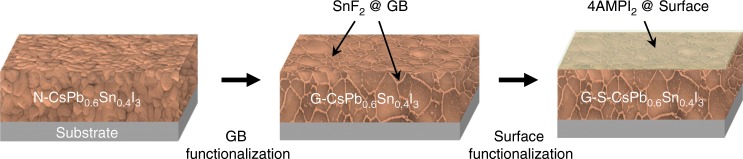
Stabilization approach for CsPb_0.6_Sn_0.4_I_3_ perovskites. Schematic illustration of interface functionalization of CsPb_0.6_Sn_0.4_I_3_ perovskite thin films via two steps: (i) grain-boundary (GB) functionalization with continuous SnF_2_ (derived from SnF_2_•3FACl) and (ii) additional top-surface functionalization with (4AMP)I_2_ salt.

To further enhance the stability of CsPb_0.6_Sn_0.4_I_3_ perovskite thin films, chemical functionalization of interfaces is performed using a combination of two previously reported approaches. The first approach is the functionalization of grain boundaries by incorporating SnF_2_•3FACl additive in the solution processing of CsPb_0.6_Sn_0.4_I_3_ perovskite thin films. This is expected to result in the continuous decoration of grain boundaries with anti-oxidating SnF_2_ phases. The merit of SnF_2_•3FACl over pure SnF_2_ is that it not only provides uniform grain boundary coating via the SnF_2_•3FACl-to-SnF_2_ decomposition process, but also reduces grain-boundary density (larger average grain size) significantly, which was demonstrated by us previously^11^. The second approach is functionalization of surfaces, which entails spin-coating of an organic salt, (aminomethyl)piperidinium diiodide (4AMP)I_2_, on the top surface of the grain-boundary functionalized CsPb_0.6_Sn_0.4_I_3_ perovskite thin film. The large organic-molecule (4AMP)^2+^ cations are expected to not only passivate the surface defects but also form a hydrophobic encapsulation layer^29–31^. Thus, the simultaneous functionalization of all interfaces in thin films (see Fig. 2) is expected to protect CsPb_0.6_Sn_0.4_I_3_ perovskite grains at the nanoscale, mitigating the environmental degradation significantly^32^. In the following discussion, the low-bandgap CsPb_0.6_Sn_0.4_I_3_ perovskites made without any functionalization, with only grain-boundary functionalization, and with both grain-boundary and surface functionalization are denoted as N-CsPb_0.6_Sn_0.4_I_3_, G-CsPb_0.6_Sn_0.4_I_3_, and G-S-CsPb_0.6_Sn_0.4_I_3_, respectively.

**Fig. 3:**
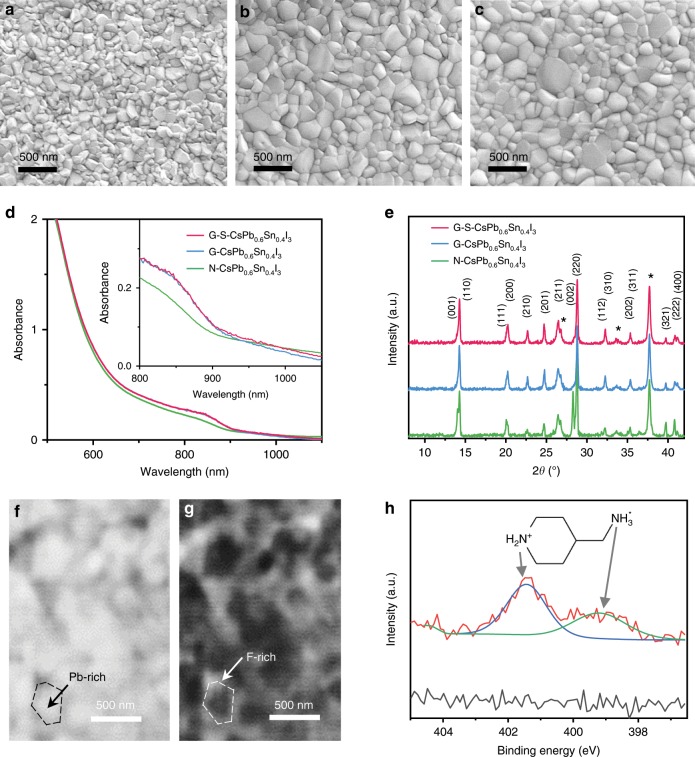
Characterizations of CsPb_0.6_Sn_0.4_I_3_ perovskites. SEM micrographs of CsPb_0.6_Sn_0.4_I_3_ thin films: **a** N-CsPb_0.6_Sn_0.4_I_3_, **b** G-CsPb_0.6_Sn_0.4_I_3_, and **c** G-S-CsPb_0.6_Sn_0.4_I_3_. **d** UV-vis absorption spectra of N-CsPb_0.6_Sn_0.4_I_3_, G-CsPb_0.6_Sn_0.4_I_3_, and G-S-CsPb_0.6_Sn_0.4_I_3_ thin films. **e** XRD patterns of N-CsPb_0.6_Sn_0.4_I_3_, G-CsPb_0.6_Sn_0.4_I_3_, and G-S-CsPb_0.6_Sn_0.4_I_3_ thin films. The FTO substrate XRD peak marked by ‘*’. X-ray ptychography reconstructed-image of the G-CsPb_0.6_Sn_0.4_I_3_ thin film. **f** Phase-contrast inverted grain distribution and **g** phase-contrast enhanced grain-boundary regions. **h** XPS spectra of CsPb_0.6_Sn_0.4_I_3 _perovskite thin films with and without (4AMP)I_2_ surface treatment.

Figure 3a, b shows top-view scanning electron microscopy (SEM) images of N-CsPb_0.6_Sn_0.4_I_3_ and G-CsPb_0.6_Sn_0.4_I_3_ perovskite thin films. The addition of SnF_2_∙3FACl increases the average grain size from 120 to 180 nm, as measured using image analyses (Supplementary Fig. 7). The XRD patterns in Fig. 3e indicate enhanced crystallinity and (110) texture in the G-CsPb_0.6_Sn_0.4_I_3_ thin film. This correspondingly increases light absorption, as shown in the UV-vis spectra (Fig. 3d). The enhancement of the microstructure and the optical properties can be attributed to two factors: (i) FACl component reduces grain-boundary density and (ii) the remaining SnF_2_ helps reduce Sn-defects within the grains in situ^11^. The SEM image and XRD pattern of a G-S-CsPb_0.6_Sn_0.4_I_3_ perovskite thin film are shown in Fig. 3c, e. A sequential (4AMP)I_2_ treatment does not result in an obvious effect on the overall morphology and film crystallinity, which is as expected. There is also no apparent change in the absorption characteristics of the thin film (Fig. 3d). We further analyzed the detailed microstructure and chemical composition in the G-S-CsPb_0.6_Sn_0.4_I_3_ thin film using X-ray ptychography and X-ray photoelectron spectroscopy (XPS). X-ray ptychography is a coherence-based X-ray technique that combines the power of scanning X-ray microscopy with coherent diffractive imaging. This allows for the multidimensional rendering of internal states (e.g. microstructure and morphology) of complex materials by utilizing both absorption and phase contrast for visualization^33^. Figure 3f, g shows two-dimensional transmission-mode X-ray ptychography reconstructed images of a G-CsPb_0.6_Sn_0.4_I_3_ perovskite thin film, where two regions with clearly distinct contrasts are observed. Heavy-element (Pb) and light-element (F) rich phases are discernable at grain and grain-boundary regions, respectively. In Figure 3h, the N 1*s* core-level XPS spectrum of the CsPb_0.6_Sn_0.4_I_3_ perovskite thin film with (4AMP)I_2_ surface treatment is shown, where two N 1*s* peaks are observed at 399.20 and 401.43 eV that are respectively assigned to -NH_3_ and -NH_2_-^34^. This confirms the formation of a thin layer of organic (4AMP)^2+^ cations on the top surface of G-S-CsPb_0.6_Sn_0.4_I_3_ perovskite thin films. More careful analyses in Supplementary Figs. 8–10 show no obvious traces of incorporation of FA^+^, Cl^−^, or (4AMP)^2+^ within the perovskite lattice, which attests to the all-inorganic nature of the G-S-CsPb_0.6_Sn_0.4_I_3_ thin films.

### Properties and stability of CsPb_0.6_Sn_0.4_I_3_ perovskites

**Fig. 4:**
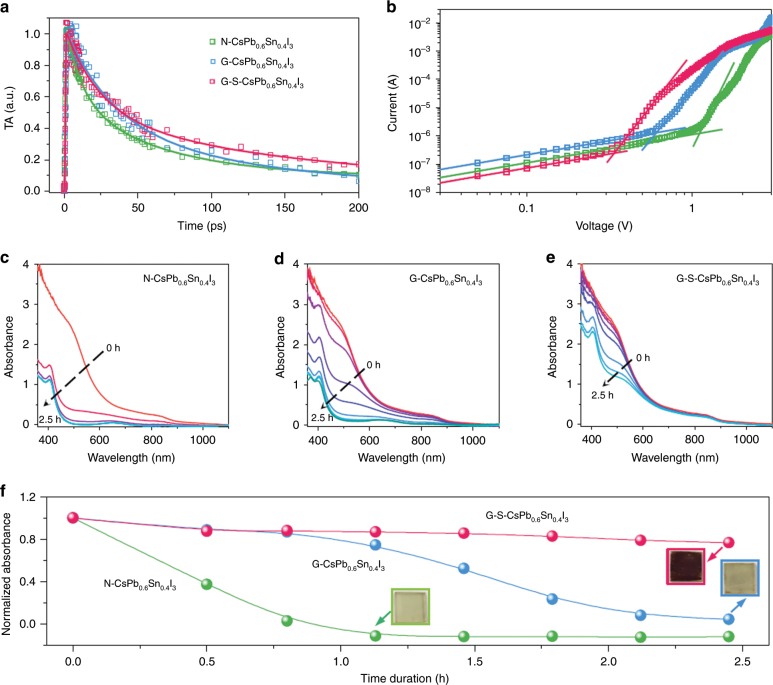
Physical properties and chemical stability of CsPb_0.6_Sn_0.4_I_3_ perovskites. **a** TA spectra of N-CsPb_0.6_Sn_0.4_I_3_, G-CsPb_0.6_Sn_0.4_I_3_, and G-S-CsPb_0.6_Sn_0.4_I_3_ thin films. **b** Dark current–voltage curves of capacitor-like devices using N-CsPb_0.6_Sn_0.4_I_3_, G-CsPb_0.6_Sn_0.4_I_3_, and G-S-CsPb_0.6_Sn_0.4_I_3_ thin films. UV-vis spectra evolution of thin films stored controlled ambient conditions (RT, 80% RH). **c** N-CsPb_0.6_Sn_0.4_I_3_, **d** G-CsPb_0.6_Sn_0.4_I_3_, and **e** G-S-CsPb_0.6_Sn_0.4_I_3_ thin films. **f** Absorbance (at 800 nm) variation as a function of storage duration. Insets are corresponding photographs of the thin films.

Figure 4a shows the transient absorption (TA) kinetics of N-CsPb_0.6_Sn_0.4_I_3_, G-CsPb_0.6_Sn_0.4_I_3_, and G-S-CsPb_0.6_Sn_0.4_I_3_ thin films. The kinetics were fitted using empirical biexponential functions with two time-constants *τ*_1_ and *τ*_2_ (see Supplementary Table 1 for fitting parameters), which could be related to surface and bulk recombination rates, respectively. Transient photoluminescence (TRPL) decay kinetics were also acquired, again showing an increase in the PL lifetimes with grain-boundary and surface functionalization. In order to estimate the overall trap densities, dark current–voltage (*I–V*) curves of capacitor-like devices were measured using N-CsPb_0.6_Sn_0.4_I_3_, G-CsPb_0.6_Sn_0.4_I_3_, and G-S-CsPb_0.6_Sn_0.4_I_3_ thin films. The capacitor-like device architecture is shown schematically in Supplementary Fig. 11. The trap-filled limited voltages (*V*_TFL_) were determined from the *I–V* curves, showing 1.11V, 0.61V, and 0.34V for N-CsPb_0.6_Sn_0.4_I_3_. G-CsPb_0.6_Sn_0.4_I_3_, and G-S-CsPb_0.6_Sn_0.4_I_3_, respectively (Fig. 4b). The trap densities, *n*_trap_, are estimated using the equation: $n_{{\mathrm{trap}}} = \frac{{2\varepsilon _0\varepsilon _{\mathrm{r}}V_{{\mathrm{TFL}}}}}{{ed^2}}$, where *e* is the elementary charge, *d* the film thickness, *ε*_r_ the relative dielectric constant, and *ε*_0_ the vacuum permittivity. Thus, the estimated trap densities for N-CsPb_0.6_Sn_0.4_I_3_, G-CsPb_0.6_Sn_0.4_I_3_, and G-S-CsPb_0.6_Sn_0.4_I_3_ are 5.50 × 10^16^, 3.02 × 10^16^, and 1.68 × 10^16^ cm^−3^, respectively. These results further confirm the beneficial effects of grain-boundary and surface passivation.

The evolution of UV-vis spectra of N-CsPb_0.6_Sn_0.4_I_3_, G-CsPb_0.6_Sn_0.4_I_3_, and G-S-CsPb_0.6_Sn_0.4_I_3_ thin films was measured as a function of storage duration under controlled ambient conditions (RT, 80% RH), and are shown in Fig. 4c–e. The absorption of N-CsPb_0.6_Sn_0.4_I_3_ thin film decreases very rapidly upon ambient exposure. After 2.5 h, the film is completely bleached, which is due to the degradation of the CsPb_0.6_Sn_0.4_I_3_ perovskite (β phase) into the CsPb_0.6_Sn_0.4_I_3_ nonperovskite (δ phase), as revealed by XRD (Supplementary Fig. 12). Such degradation is slowed down significantly in G-CsPb_0.6_Sn_0.4_I_3_ and G-S-CsPb_0.6_Sn_0.4_I_3_ thin films. With the full functionalization, the latter perovskite film retains all the absorption for 2.5 h. Figure 4f plots the absorbance at 800 nm as a function of storage time for the three thin films, where the stabilization effect of grain boundary and surface functionalization is seen clearly. In addition, the G-S-CsPb_0.6_Sn_0.4_I_3_ thin film again shows the best thermal stability, as shown in Supplementary Fig. 13.

### Device performance of CsPb_0.6_Sn_0.4_I_3_ perovskites

**Fig. 5:**
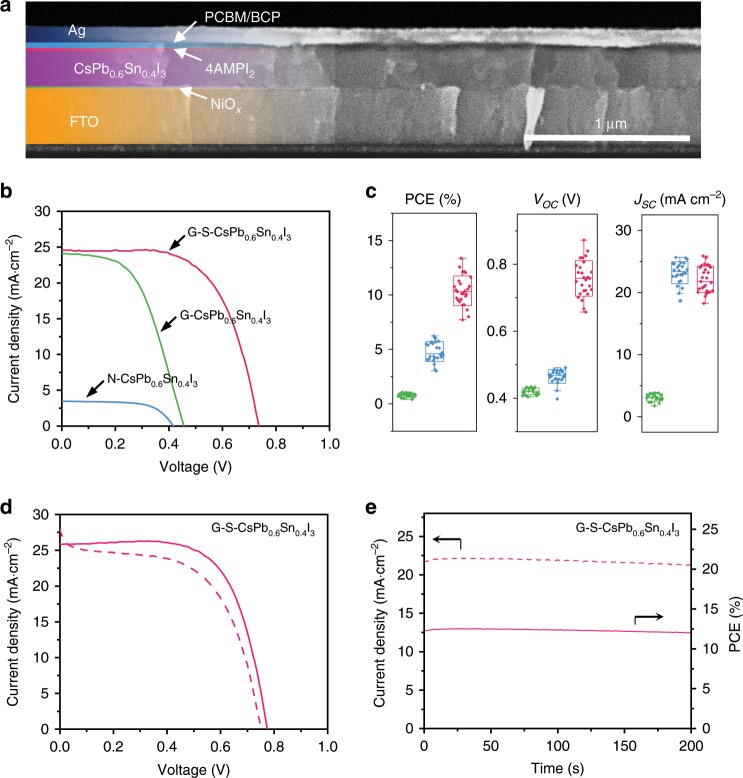
Photovoltaic performance of CsPb_0.6_Sn_0.4_I_3_ perovskite solar cells. **a** SEM cross-sectional image of a typical CsPb_0.6_Sn_0.4_I_3_-based PSC. **b** Typical *J–V* curves of perovskite solar cells based on N-CsPb_0.6_Sn_0.4_I_3_, G-CsPb_0.6_Sn_0.4_I_3_, and G-S-CsPb_0.6_Sn_0.4_I_3_ thin films. **c** The corresponding *J–V* parameter (PCE, *V*_OC_, *J*_SC_) statistics of 20–30 devices based on boxplots. **d** *J–V* hysteresis (reverse scan: solid line; forward scan: dashed line) and **e** corresponding stabilized current and power outputs of the champion G-S-CsPb_0.6_Sn_0.4_I_3_-based PSC at maximum power point. The extracted *J-V* parameters from b-c are shown in Table 1.

PSC devices were fabricated to evaluate the photovoltaic performance. The inverted planar architecture of FTO/NiO_*x*_/perovskite/PCBM/BCP/Ag is used, which is typically used for Sn-containing PSCs^14,35,36^. A cross-sectional SEM image of the device is shown in Fig. 5a, where each layer is delineated clearly. Figure 5b compares typical current density–voltage (*J*–*V*) responses from PSCs based on N-CsPb_0.6_Sn_0.4_I_3,_ G-CsPb_0.6_Sn_0.4_I_3_, or G-S-CsPb_0.6_Sn_0.4_I_3_ thin films. N-CsPb_0.6_Sn_0.4_I_3_ PSC shows relatively low PCE of 0.92% with short-circuit current density (*J*_SC_), 3.47 mA cm^−2^ and open-circuit voltage (*V*_OC_) of 0.416 V and fill factor (FF) of 0.641. After grain-boundary functionalization, *J*_SC_ and *V*_OC_ are boosted to 24.07 mA cm^−2^ and 0.457 V, respectively, leading to an improved PCE of 5.68%. For the G-S-CsPb_0.6_Sn_0.4_I_3_ PSC, PCE of 11.28% is achieved, with *J*_SC_ of 24.57 mA cm^−2^, *V*_OC_ of 0.735 V, and FF of 0.624. Figure 5c shows the PCE statistics, indicating good reproducibility of the PCE improvement with both grain-boundary and surface functionalization. Figure 5d shows *J–V* curves of the champion PSC based on G-S-CsPb_0.6_Sn_0.4_I_3_. This device exhibits typical hysteresis with 13.37% PCE in reverse scan and 11.39% PCE in forward scan. Maximum-power-point current/PCE output was thus monitored (Fig. 5e), which shows stabilized PCE of 12.51%. Compared with high-performance low-bandgap organic–inorganic hybrid PSCs, our PSCs exhibit relatively lower FF, which may be due to more significant carrier recombination. In this regard, a systematic study about the carrier mobility and carrier diffusion lengths of our films will be performed in the future for understanding and improving the carrier dynamics.

**Table 1:** *J–V* parameters of CsPb_0.6_Sn_0.4_I_3_ perovskite solar cells.

Sample	*J*_SC_ (mA cm^−2^)	*V*_OC_ (V)	FF	PCE (%)
N-CsPb_0.6_Sn_0.4_I_3_	3.47 ± 0.36	0.416 ± 0.018	0.641 ± 0.017	0.92 ± 0.14
G-CsPb_0.6_Sn_0.4_I_3_	24.07 ± 1.60	0.457 ± 0.034	0.517 ± 0.052	5.68 ± 0.55
G-S-CsPb_0.6_Sn_0.4_I_3_	24.57 ± 1.30	0.735 ± 0.137	0.624 ± 0.064	11.28 ± 2.09
G-S-CsPb_0.6_Sn_0.4_I_3_ (R)^a^	25.87	0.774	0.667	13.37
G-S-CsPb_0.6_Sn_0.4_I_3_ (F)^a^	26.11	0.749	0.583	11.39

Photovoltaic performance parameters of CsPb_0.6_Sn_0.4_I_3_ perovskite solar cells without any functionalization (N-CsPb_0.6_Sn_0.4_I_3_), with only grain-boundary functionalization (G-CsPb_0.6_Sn_0.4_I_3_), and with both grain-boundary and surface functionalization (G-S-CsPb_0.6_Sn_0.4_I_3_). The parameters are obtained based on Fig. 5b–d

^a^Champion cell; R = reverse scan and F = forward scan

**Fig. 6:**
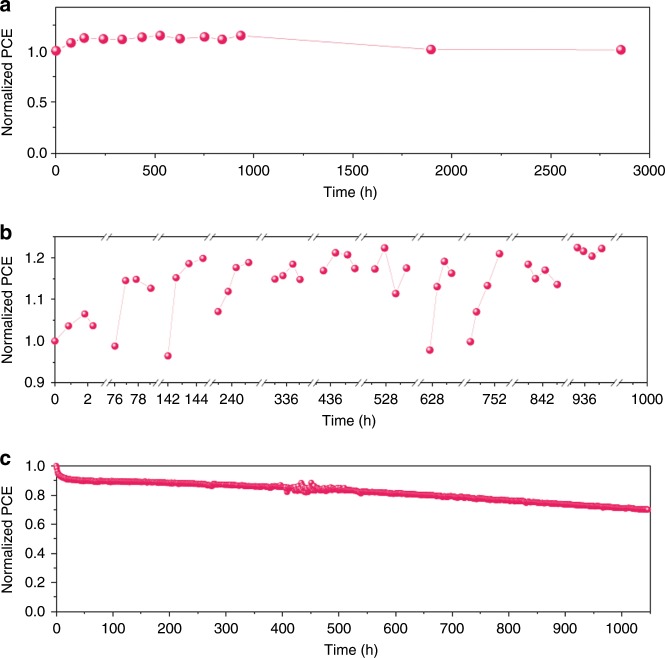
Long-term stability of CsPb_0.6_Sn_0.4_I_3_ perovskite solar cells. Normalized PCE of a G-S-CsPb_0.6_Sn_0.4_I_3_-based PSC (unencapsulated) as a function of time under different conditions: **a** shelf storage in dry N_2_ atmosphere at RT, **b** controlled exposure to dry ambient environment (RT, below 20% RH) periodically, and **c** continuous operation under one-sun intensity illumination (nitrogen atmosphere at around 45 °C).

The long-term stability of the G-S-CsPb_0.6_Sn_0.4_I_3_-based PSCs was evaluated. First, the shelf life of the PSCs upon storage in dry N_2_ atmosphere at RT was tested. As shown in Fig. 6a, after 2800 h, no obvious PCE degradation is observed. The stability of PSCs exposed to controlled ambient conditions was also examined (Fig. 6b). Here the PSC was exposed to the ambient atmosphere (RT, below 20% RH) for 2–3 h every 3–4 days. Interestingly, upon ambient exposure, the PCE gradually increases in the initial stage, and reaches a PCE plateau. A similar trend was observed within every cycle of ambient exposure, and eventually the overall device PCE was surprisingly increased after a total time of 1000 h. This attests to the stability of the G-S-CsPb_0.6_Sn_0.4_I_3_-based PSC for deployment in the real world where the environmental humidity usually changes in a periodic manner. Finally, the operational stability of this PSC was tested during continuous operation under one-sun illumination (nitrogen atmosphere, ~45 °C). The conditions for such tests are chosen to be similar to those in the literature^17,37–39^. As seen in Fig. 6c, after 1000 h, the PSC shows very high PCE retention of 71%. *T*_80_ lifetime of this PSC is 653 h, and extended stability test shows T70 lifetime of 1045 h. It is worth noting this is the first report on 1000-h operation stability of low-bandgap PSCs. Stability test results for more devices are included in Supplementary Fig. 14 to demonstrate the reproducibility. Prior to this study, Tong et al.^17^ reported 220-h *T*_80_ lifetime. Other related studies report only short-term or long-term shelf stability^11,12,15,16,40^. See Supplementary Table 2 for a detailed compilation of these stability results. The device stability during storage in the nitrogen-filled glovebox was further evaluated at a temperature of 85 °C which is typically used in the literature^41^ (Supplementary Fig. 15), which shows 77% PCE retention after 100 h. In this context, this study reports low-bandgap PSCs with the most comprehensive stability results and especially the best operational stability so far. This demonstrates clearly the merit of the inorganic low-bandgap PSCs over their hybrid organic–inorganic counterparts.

## Discussion

All the above results confirm CsPb_0.6_Sn_0.4_I_3_ as a promising low-bandgap halide perovskite candidate for PSCs. The interfaces (grain boundaries and surfaces) in this perovskite play a key role in determining its physical properties, chemical stability, and device function. With rational functionalization of the interfaces, inorganic CsPb_0.6_Sn_0.4_I_3_ perovskite can deliver high PCE in PSCs, on a par with those reported for hybrid organic–inorganic low-bandgap perovskites. Regarding the stability, the all-inorganic nature of CsPb_0.6_Sn_0.4_I_3_ perovskite offers inherently superior stability compared to their hybrid counterparts. In addition, the formation energy of Sn-defects in inorganic perovskites is smaller than that in hybrid perovskites due to the smaller ionic size of Cs^+^, and, thus, stronger antibonding of Sn 5*s* with I 5*p*^42^. All these factors contribute to the superior long-term stability that is observed under various accelerated test conditions.

While this work provides a successful demonstration of efficient inorganic low-bandgap PSCs, the detailed mechanisms underlying the key interface functionalization remain unclear, which will require further investigation. Three major effects of interface functionalization may be at play: (i) SnF_2_ nanophases derived from SnF_2_•3FACl serve as Sn(II) sources for in situ annihilation of Sn-related defects generated during processing, storage, and operation of PSCs. (ii) The surface functionalization with (4AMP)^2+^ molecule cations may passivate the defects and prevent the bulk CsPb_0.6_Sn_0.4_I_3_ perovskite thin films from moisture ingression. We find the combination of –NH_3_^+^ and =NH_2_^+^ functional groups may be key to the effective surface functionalization, as the replacement of (4AMP)I_2_ with either piperazine (with two = NH_2_^+^ groups) iodide or phenylethylammonium (with only –NH_3_^+^) iodide leads to lower PCE (see *J*–*V* curves in Supplementary Fig. 16). The atomistic origin of the (4AMP)I_2_ passivation effect needs to be studied further, which may guide the design of even more effective surface functionalization agents. (iii) The (4AMP)I_2_ surface functionalization also contributes to the change of energy level which facilitates the carrier injection at the perovskite/electron-transporting-layer interface (see UPS analyses in Supplementary Fig. 17). This is typical for interface engineering strategy in PSCs^41,43^. We have also observed that both G-CsPb_0.6_Sn_0.4_I_3_ and G-S-CsPb_0.6_Sn_0.4_I_3_ thin films still show rapid oxidation of Sn^2+^ to Sn^4+^ in the ambient environment based on preliminary XPS results (Supplementary Fig. 18). Therefore, we envision that a functionalizing molecular agent that can be more resistant to oxygen may contribute to more efficient and stable CsPb_0.6_Sn_0.4_I_3_-based PSC devices.

In addition, we have observed a promising, but unusual, feature of device PCE enhancement under controlled, periodic exposure to the ambient air, which may be related to further passivation of various interfaces (charge-transport-layer/perovskite interfaces, grain boundaries) induced by ambient air. This indicates that by tailoring the atmosphere-perovskite or atmosphere-device interactions, the device PCE and stability may be further enhanced. It is recognized that it will be important for evaluating the stability of the reported low-bandgap PSC devices under standardized testing conditions that are just being established^44^.

Based on all our results, we envision the low-bandgap inorganic perovskite of CsPb_0.6_Sn_0.4_I_3_ and its functionalization reported in this study pointing to a research direction for developing stable PSCs with PCEs approaching the S–Q limit. These results may also have implications on the development of Sn-based Pb-free PSCs, as well as perovskite–perovskite tandem photovoltaics.

## Methods

### Raw chemicals

Dimethylformamide (DMF), dimethyl sulfoxide (DMSO), chlorobenzene (CBE), isopropanol (IPA), 4-(aminomethyl)piperdine (4AMP), hydroiodic acid (HI; 57 wt% in H_2_O), tin(II) iodide (SnI_2_), tin(II) fluoride (SnF_2_), nickel(II) acetate tetrahydrate (Ni(COOCH_3_)_2_∙4H_2_O), and copper(II) acetate monohydrate (Cu(COOCH_3_)_2_∙H_2_O) were purchased from Sigma-Aldrich (USA). Cesium iodide (CsI) and lead iodide (PbI_2_) were purchased from Alfa-Aesar (USA). Formamidinium chloride (FACl) was purchased from GreatCell Solar (Australia). Phenyl-C61-butyric acid methyl ester (PCBM) and bathocuproine (BCP) were purchased from 1-Material (Canada). All raw chemicals were used without further purification.

### Preparation of precursors and thin films

(4AMP)I_2_ and ‘HPbI_3_’ solids were synthesized according to methods reported in the literature^45,46^. For (4AMP)I_2_, 2.0 g of 4AMP was added in 20 mL of 2-propanol. While stirring, 7.5 g of HI was added. The solution was further stirred overnight at RT, which was followed by the removal of the solvent using a rotary evaporator. The resulting (4AMP)I_2_ powder was stored in glovebox for further use. For ‘HPbI_3_,’ 0.461 g of PbI_2_ and 0.224 g of HI were added to 0.787 g of DMF solvent to form a 40 wt% ‘HPbI_3_’ solution. The ‘HPbI_3_’ solution was heated at 80 °C in CBE vapor environment overnight. During this heat treatment, the CBE molecules diffuse into the ‘HPbI_3_’/DMF solution and reduce the solubility of ‘HPbI_3_’. Light-yellow needle-like ‘HPbI_3_’ powder particles were formed and then collected. Finally, the ‘HPbI_3_’ crystals were dried at RT for 48 h under vacuum. Note that the exact composition of ‘HPbI_3_’ is still under debate, and the effect of ‘HPbI_3_’ use on the final perovskite composition remains unclear^28,47^. In this study, the use of ‘HPbI_3_’ significantly enhances the overall inorganic perovskite stability, without influencing the bandgap and crystal-structure parameters. For synthesizing SnF_2_•3FACl, the method reported in our previous work was used^11^. Briefly, SnF_2_ and FACl powders are co-dissolved in DMF in molar ratio 1:3, followed by recrystallization and harvesting of this compound.

For making NiO_*x*_ precursor solution, 125 mg of Ni(COOCH_3_)_2_∙4H_2_O was dissolved in 5 mL of ethanol with 30 µL of ethanolamine, and 20 mg of Cu(COOCH_3_)_2_∙H_2_O dissolved in 1 mL of ethanol. Both solutions were then stirred on hotplate at 50 °C for 30 min. The final NiO_*x*_ precursor solution was obtained by mixing 26.5 µL of copper-salt solution and 5 mL of nickel-salt solution. For making (4AMP)I_2_ solutions, a saturated (4AMP)I_2_ IPA solution was first prepared, which was then diluted fivefold.

For preparing N-CsPb_0.6_Sn_0.4_I_3_ thin films, typically, a 0.7 M N-CsPb_0.6_Sn_0.4_I_3_ solution was first prepared. Here, 0.1091 g of CsI, 0.0581 g of PbI_2_, 0.0799 g of ‘HPbI_3_’, and 0.0625 g of SnI_2_ were co-dissolved in a mixed solvent of 71 µL of DMSO and 500 mg of DMF. The N-CsPb_0.6_Sn_0.4_I_3_ thin films were prepared by spin-coating the solution at 3000 rpm for 30 s. The as-spun thin films were heated at 60 °C for 2 min, and then 120 °C for 30 min. For the preparation of G-CsPb_0.6_Sn_0.4_I_3_ thin films, 8.4 mg of SnF_2_•3FACl was added in the 0.7 M N-CsPb_0.6_Sn_0.4_I_3_ solution to form the precursor solution. The same spin-coating and annealing conditions were used for preparing N-CsPb_0.6_Sn_0.4_I_3_ thin films. For making G-S-CsPb_0.6_Sn_0.4_I_3_ thin films, a post-treatment step was used, which involved the spin-coating of the (4AMP)I_2_ solution at 2000 rpm for 30 s.

### Materials characterization

Laboratory XRD was performed using a high-resolution diffractometer (D8 Advance, Bruker; Germany) with Cu K_α_ radiation. XRD pattern and crystal-structure information was refined using the Rietveld method with MAUD software^48^. The *sig* and *R*_wp_ parameters were found to be 1.60 and 5.44, respectively. UV-vis spectra were obtained using a spectrophotometer (UV-2600, Shimadzu; Japan). The microstructures of the thin films were observed using a scanning electron microscope (Thermo Scientific Quattro S ESEM; USA). XPS and UPS systems (5600, PHI; USA) were used to acquire the XPS and UPS spectra. The instrument utilized a monochromated Al K_α_ source for X-ray radiation at 1486.7 eV, and a UVS 40A2 (PREVAC, Poland) UV source and UV40A power supply provided by He 1α for UPS at 21.22 eV. Femtosecond TA measurements were performed with a 800-nm, 35-fs amplified Ti:sapphire laser at a repetition rate of 2 kHz. An optical parametric amplifier was used to produce the pump pulses with tunable wavelengths, which were reduced in repetition rate down to 1 kHz with a mechanical chopper. A portion of the amplifier output was directed onto a 12-mm-thick YAG crystal to produce broadband near-infrared probe pulses at 2 kHz. FTIR spectra were obtained on a spectrometer (4100, Jasco Instruments, USA). The samples for the FTIR measurements were prepared by scratching the thin films from the substrates. For time-resolved photoluminescence (TRPL) measurements, the samples were excited at 405 nm with a picosecond diode laser, and the emitted photons were captured using time-correlated single-photon-counting electronics (PicoQuant; Germany). Time-integrated photoluminescence spectra were excited at 405 nm also and measured with a CCD camera. Synchrotron-based X-ray ptychography was performed at the Velociprobe^49^ located at 2-ID-D beamline at the Advanced Photon Source (APS) in Argonne National Laboratory. An 8.8-keV monochromatic X-ray beam was focused by a Fresnel zone plate with an outer zone width of 50 nm. The sample was placed around 500 μm downstream of the focusing position of the zone plate, with an illumination size of about 1.2 μm. As the sample was raster-fly scanned, a Dectris Eiger 500K hybrid pixel array detector (1.92 m downstream of the sample) collected forward scattering patterns at a continuous frame rate of 100 Hz. Those scattering patterns were reconstructed into real-space images with 14 nm pixels by a GPU-based code^50^.

### DFT calculation

DFT-based first-principles calculations were conducted using the plane-wave basis in Cambridge Sequential Total Energy Package (CASTEP)^51^ code. The exchange-correlation functional was used by Perdew–Burke–Ernzerhof generalized gradient approximation (PBE-GGA)^52^. The ultrasoft pesudopotentials^53^ within the frozen core approximation were employed, i.e., Cs 5s^2^ 5p^6^ 6s^1^, Pb 5d^10^ 6s^2^ 6p^2^, Sn 5s^2^ 5p^2^, and I 5s^2^ 5p^5^. The Monkhorst-Pack type *k*-point was 3 × 3 × 2. The kinetic energy cutoff was set to 290 eV. The geometry optimization thresholds for energy change, maximum force, maximum stress, and maximum displacement between cycles were set to 5 × 10^−6^ eV atom^−1^, 0.01 eV Å^−1^, 0.02 GPa, and 5 × 10^−4^ Å, respectively.

### Device fabrication

Fluorinated-tin oxide (FTO)-coated glass substrates were cleaned by sequentially washing with detergent, deionized water, ethanol, acetone, and IPA. Before use, the FTO-glass substrates were treated with UV-ozone for 20 min. Subsequently, a NiO_*x*_ compact layer was deposited by spin-coating the precursor solution at 3000 rpm for 60 s, followed by annealing at 400 °C for 60 min in air. Next, the perovskite thin film was deposited according to the procedure described above. PCBM (using a solution of 23 mg mL^−1^ in CBE) and BCP (a saturated solution in IPA) were then sequentially spin-coated onto the perovskite thin films at 3000 rpm for 30 s and 1500 rpm for 30 s, respectively. Finally, 100-nm Ag layer was thermally evaporated with a shadow mask. Except the steps of FTO-glass cleaning and NiO_*x*_ deposition, all device fabrication steps were conducted in a N_2_-filled glovebox with both O_2_ and H_2_O levels below 0.1 ppm.

### Solar cell performance testing

The *J*–*V* characteristics of PSCs were measured using the 2400 SourceMeter under simulated one-sun AM1.5G 100 mW cm^−2^ intensity (Sol3A Class AAA, Oriel, Newport, USA) in air (RT, 40 to 60% RH), using both reverse (from *V*_OC_ to *J*_SC_) and forward (from *J*_SC_ to *V*_OC_) scans with a step size of 0.0056 V and a delay time of 10 ms. The maximum-power output stability of PSCs was measured by monitoring the *J* output at the maximum power-point bias (deduced from the reverse-scan *J*–*V* curves) using the 2400 SourceMeter. A typical active area of 0.105 cm^2^ was defined using a non-reflective mask for the *J*–*V* measurements. The stable PCE output was calculated using the following relation at the maximum power point: PCE (%) = *J* (mA cm^−2^) × *V* (V). A shutter was used to control the one-sun illumination on the PSC.

### Long-term stability testing

For device operational stability test, unencapsulated PSCs were placed in a sealed cell holder with a transparent quartz cover. A continuous flow of N_2_ gas was passed through the holder to minimize the water and oxygen content in the atmosphere. *J–V* curves were measured every 12 h. The temperature of the devices was maintained at around 45 °C under continuous one-sun-intensity white-LED illumination. Between the *J–V* measurements, the PSCs were biased at the maximum-power-point voltage using a potentiostat under illumination. For device shelf stability tests, PSCs were repeatedly measured under standard testing conditions after certain durations of storage in nitrogen or air, which is described in detail in the main text.

### Reporting summary

Further information on research design is available in the Nature Research Reporting Summary linked to this article.

## Supplementary information

Supplementary Information

Reporting Summary

## Data Availability

The authors declare that the data related to this study are available upon reasonable request.
