# Supplementary material for: Sub-1.4eV bandgap inorganic perovskite solar cells with long-term stability
Source: Nat Commun. 2020 Jan 9;11:151. doi: 10.1038/s41467-019-13908-6 (PMC6952449; doi:10.1038/s41467-019-13908-6)
Supplement: Supplementary file 1 — Supplementary Information [file 41467_2019_13908_MOESM1_ESM.pdf]

Supplementary Information

Low-Bandgap Inorganic Perovskite Solar Cells with Long-Term Stability

Mingyu Hu[1,2], Min Chen[1], Peijun Guo[3], Hua Zhou,[4] Junjing Deng,[4] Yudong Yao,[4] Yi Jiang,[4] Jue Gong[1], Zhenghong Dai[1], Yunxuan Zhou[2], Feng Qian[2], Xiaoyu Chong[2], Jing Feng[2,*], Richard D. Schaller,[3,6] Kai Zhu,[5] Nitin P. Padture [1,*] Yuanyuan Zhou[1,*]

[1] School of Engineering, Brown University, Providence, Rhode Island 02912, USA

[2] Faculty of Material Science and Engineering, Kunming University of Science and Technology, Kunming 650093, China

[3] Center for Nanoscale Materials, Argonne National Laboratory, Lemont, Illinois 60439, USA

[4] Advanced Photon Sources, Argonne National Laboratory, Lemont, Illinois 60439, USA

[5] National Renewable Energy Laboratory, Golden, CO 80401, USA

[6] Department of Chemistry, Northwestern University, Evanston, Illinois 60208, USA

[*]Correspondence and requests for materials should be addressed to J.F. (email: jingfeng@kmust.edu.cn), or N.P.P. (email: nitin_padture@brown.edu), or Y.Z. (email: yuanyuan_zhou@brown.edu)

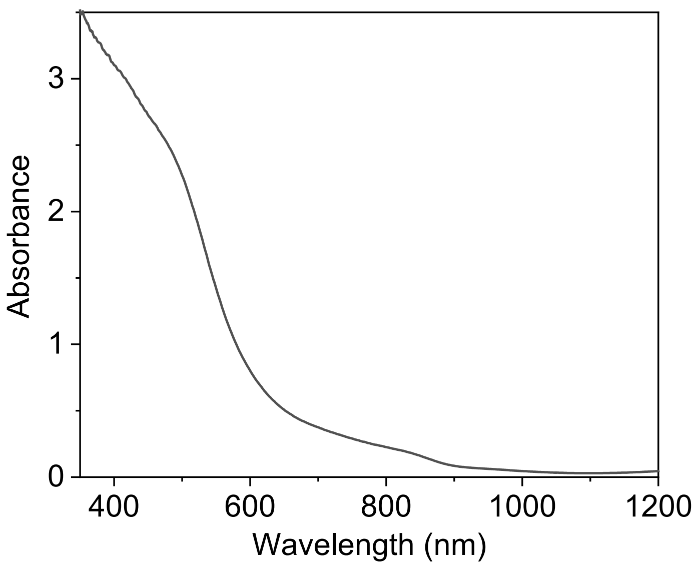

Supplementary Figure 1. UV-vis absorption spectrum of a $CsPb_{0.6}Sn_{0.4}I_3$ perovskite thin film showing full-range of light absorption from UV to IR regions.

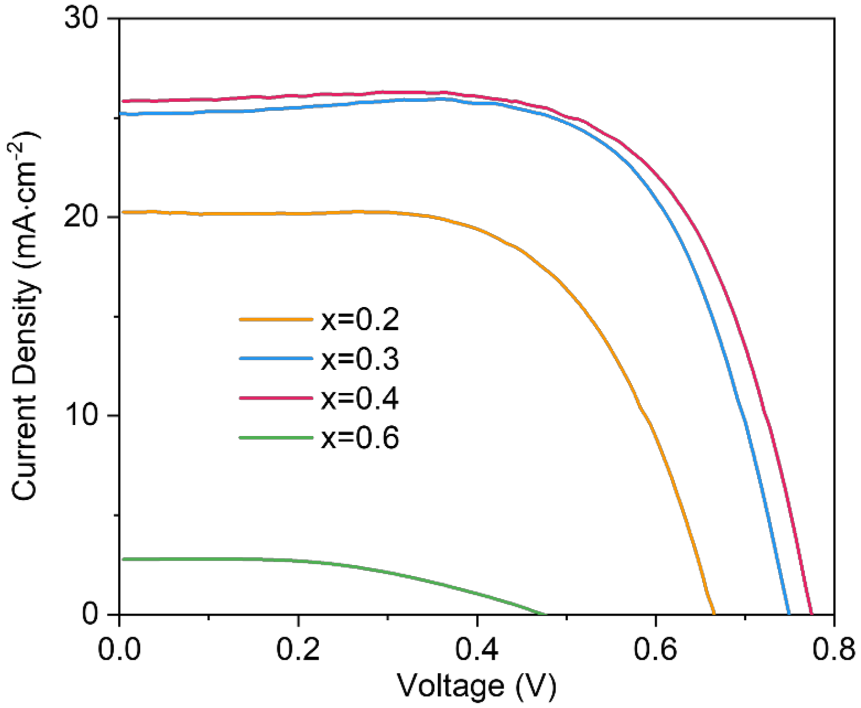

	V_{oc} (V)	J_{sc} (mA.cm^{-2})	FF	PCE (%)
x=0.2	0.731	20.29	0.617	9.160
x=0.3	0.749	25.22	0.684	12.905
x=0.4	0.774	25.87	0.678	13.366
x=0.6	0.473	2.77	0.487	0.638

Supplementary Figure 2. *J-V* curves of PSC devices based on G-S-$CsPb_{1-x}Sn_xI_3$ thin films with x=0.2, 0.3, 0.4, and 0.6. The extracted *J-V* parameters are shown in the bottom panel of the figure.

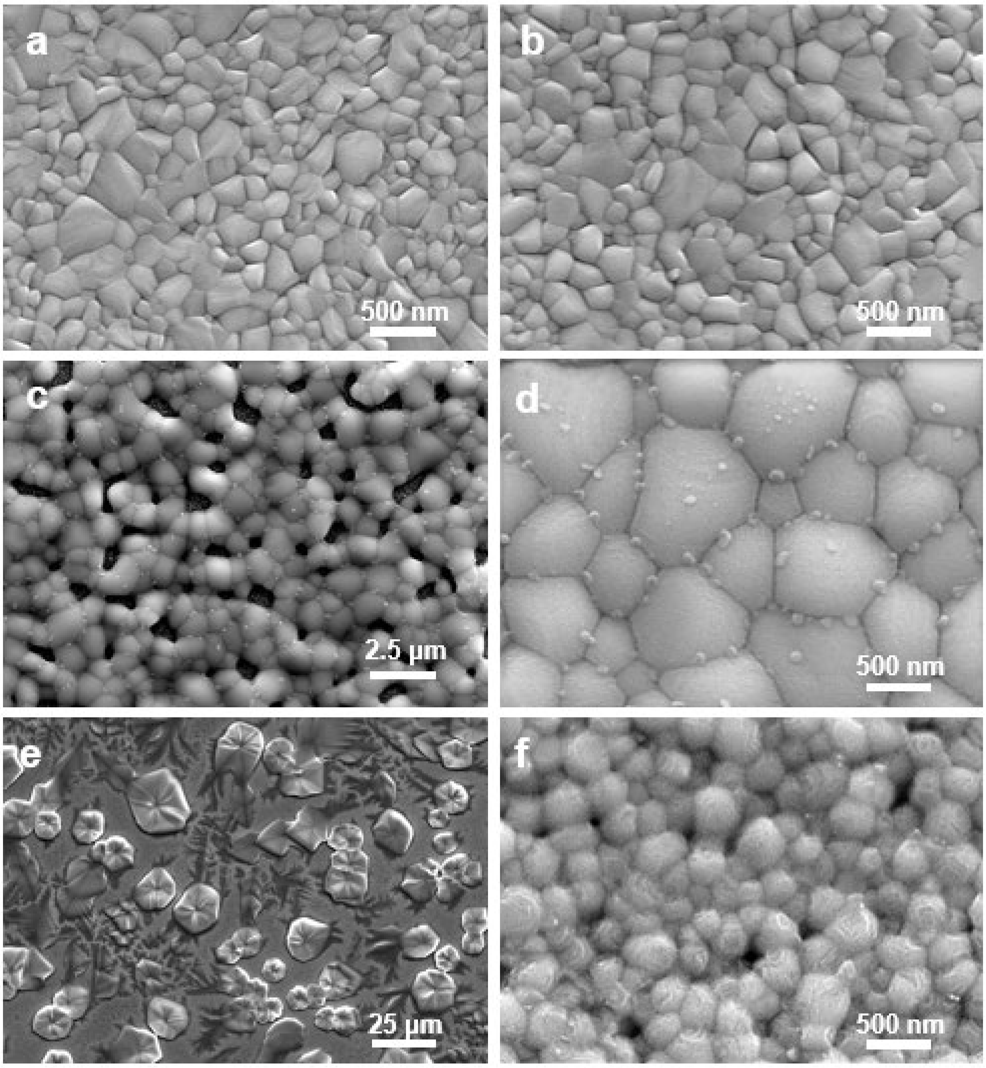

Supplementary Figure 3. SEM micrographs of low-bandgap perovskite thin films made by one-step spin-coating method with and without antisolvent treatment, respectively: (a, b) $CsPb_{0.6}Sn_{0.4}I_3$; (c, d) $(FAPbI_3)_{0.7}(CsSnI_3)_{0.3}$; and (e, f) $(FASnI_3)_{0.6}(MAPbI_3)_{0.4}$.

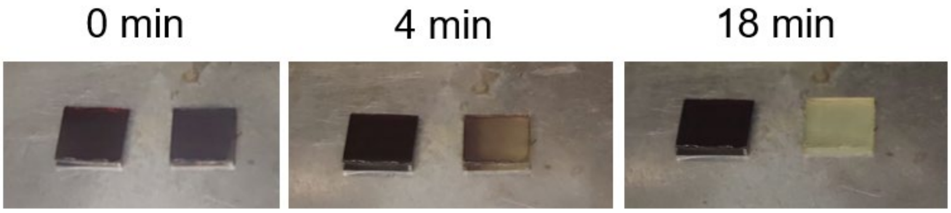

Supplementary Figure 4. Photographs of $CsPb_{0.6}Sn_{0.4}I_3$ (left) and $CsPbI_3$ (right) perovskite thin films after heating at 80 ºC in a nitrogen-filled glovebox for 0, 4, and 18 min.

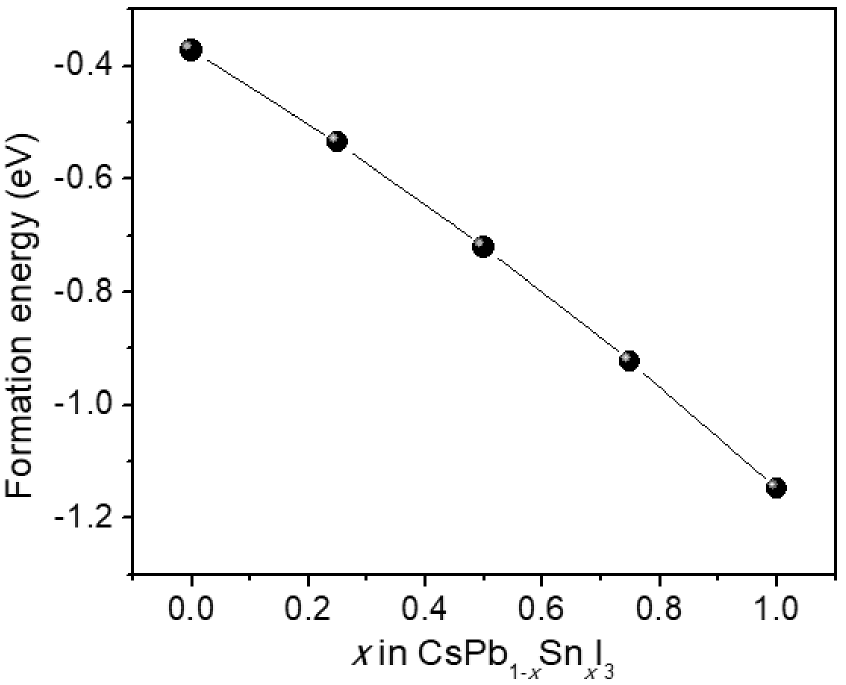

Supplementary Figure 5. Calculated formation energies of $CsPb_{1-x}Sn_xI_3$ as a function of x: $\Delta H_f = E(CsSn_xPb_{1-x}I_3) - [E(CsI) + xE(SnI_2) + (1-x)E(PbI_2)]$. The Monkhorst-Pack type k-point separation used in all calculations was 0.04 Å^{-1}, and all the other DFT calculation parameters are consistent with the calculation parameters in the main text.

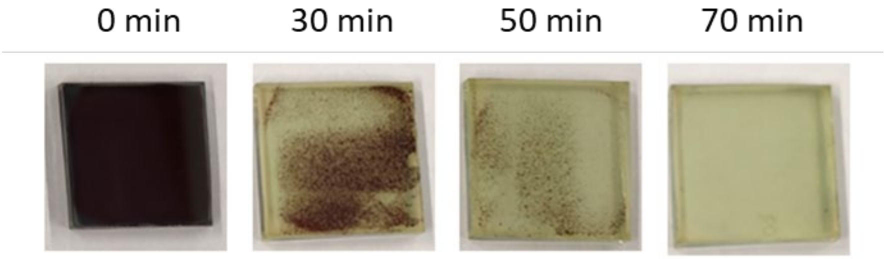

Supplementary Figure 6. Photographs showing the $CsPb_{0.6}Sn_{0.4}I_3$ perovskite thin film degrading quickly when exposed to ambient conditions with a high relative humidity (RT; 80% RH).

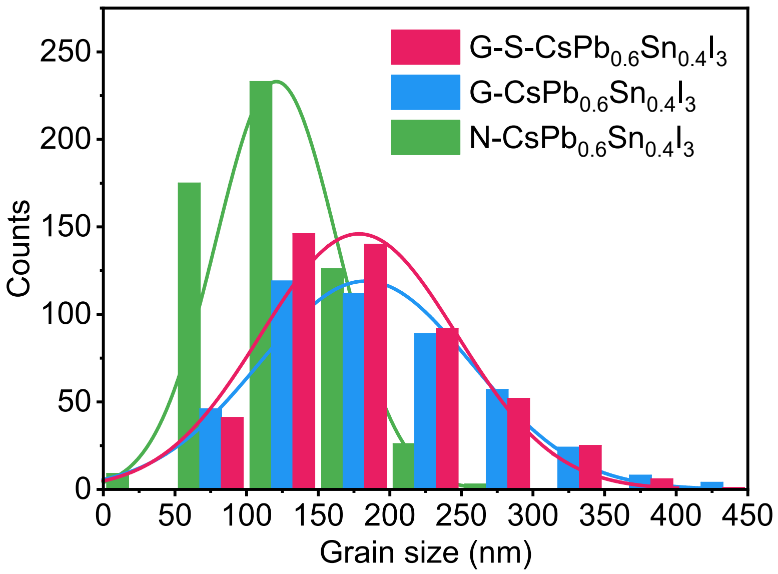

Supplementary Figure 7. Grain size distributions (based on image analyses) of $CsPb_{0.6}Sn_{0.4}I_3$ perovskite thin films: (a) N-$CsPb_{0.6}Sn_{0.4}I_3$, (b) G-$CsPb_{0.6}Sn_{0.4}I_3$, and (c) G-S-$CsPb_{0.6}Sn_{0.4}I_3$.

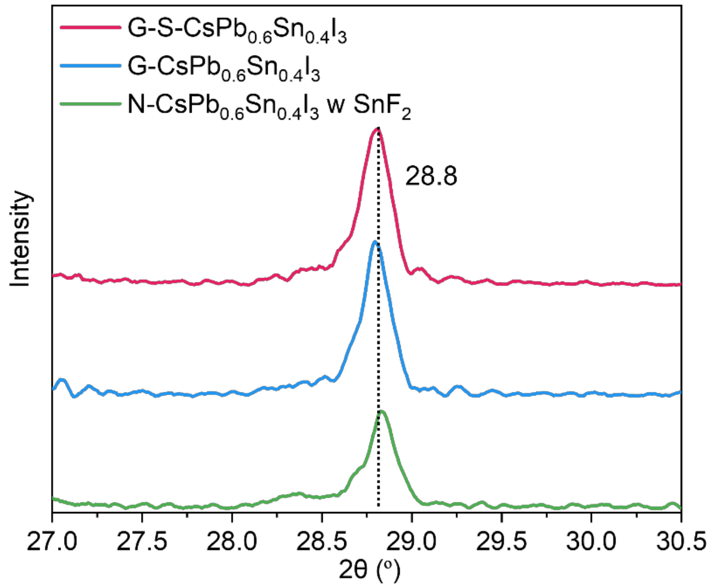

Supplementary Figure 8. XRD patterns of $CsPb_{0.6}Sn_{0.4}I_3$ thin films made with SnF_2 additive, with SnF_2•3FACl, and with both SnF_2•3FACl additive and $(4AMP)I_2$ surface treatment.

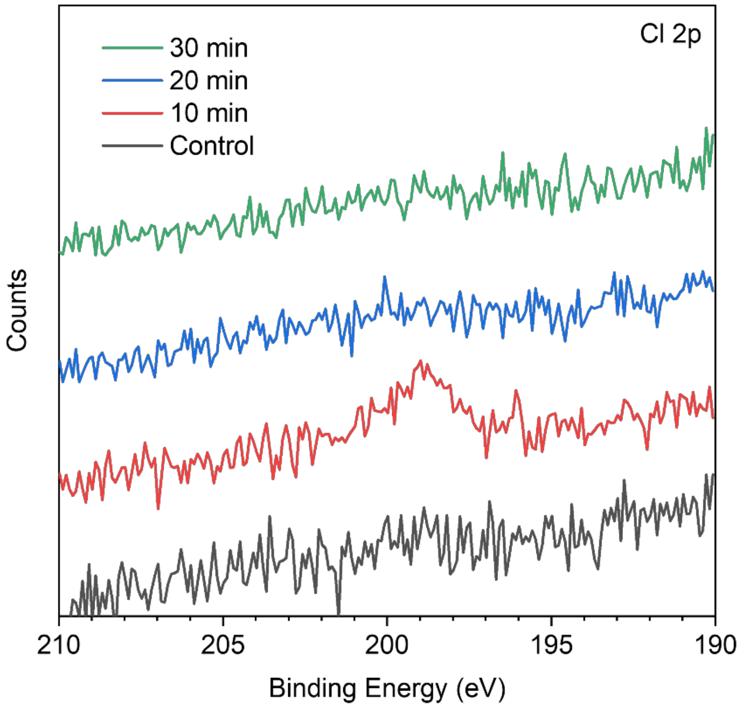

Supplementary Figure 9. XPS spectra of $CsPb_{0.6}Sn_{0.4}I_3$ thin films made with SnF_2•3FACl additive with thermal annealing at 120 °C for 10, 20, and 30 min. The XPS spectrum of $CsPb_{0.6}Sn_{0.4}I_3$ thin film made without SnF_2•3FACl additive is also presented as the control spectrum. The Cl peak (at 198.9 eV) is diminished after 20-min annealing, which indicates that FACl component is decomposed or sublimed during the annealing process.

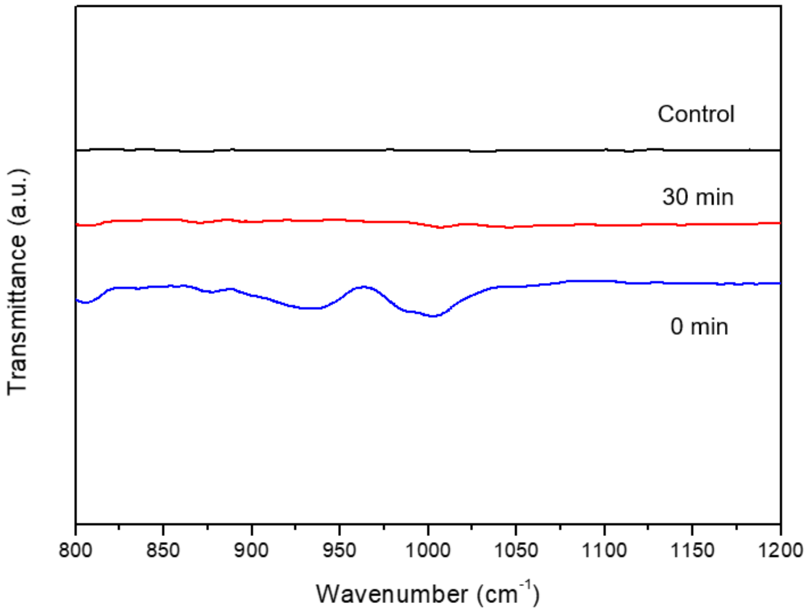

Supplementary Figure 10. FTIR spectra of $CsPb_{0.6}Sn_{0.4}I_3$ thin films made with SnF_2•3FACl additive with and without thermal annealing at 120 °C for 30 min. The FTIR spectrum of $CsPb_{0.6}Sn_{0.4}I_3$ thin film made without SnF_2•3FACl additive is also presented as the control spectrum. The valleys associated with the FA^+ component almost disappear after 30-min annealing, which further attests that FACl component is decomposed or sublimed during the annealing process.

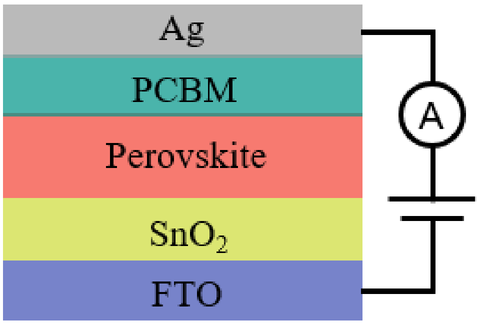

Supplementary Figure 11. Schematic illustration of the capacitor-like device architecture used for estimating the trap density.

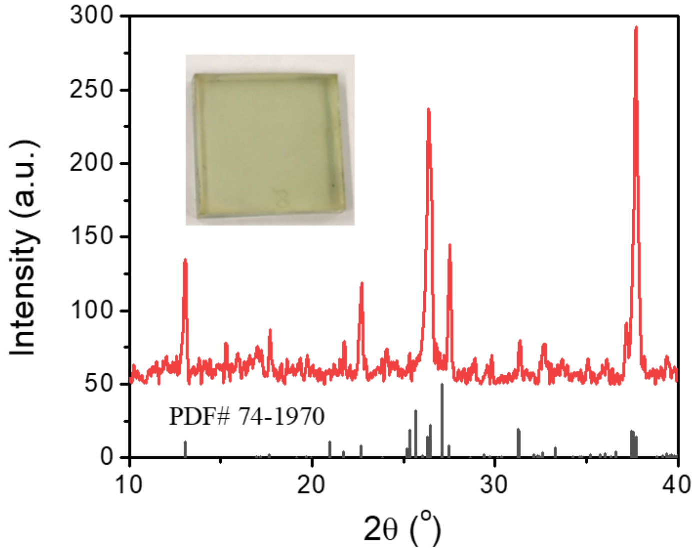

Supplementary Figure 12. XRD pattern of the degradation product of a neat $CsPb_{0.6}Sn_{0.4}I_3$ perovskite after 2.5-h exposure to ambient conditions (RT; 80% RH) confirming it to be the δ phase.

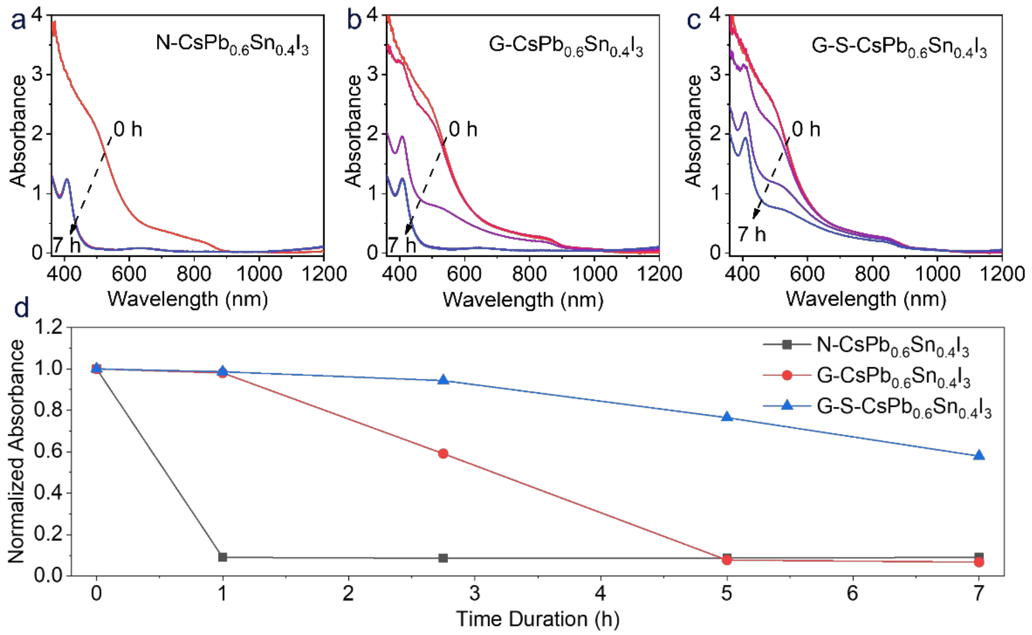

Supplementary Figure 13. Absorption spectra evolution of perovskite thin films at 180 ℃ in a nitrogen-filled glovebox: (a) N-$CsPb_{0.6}Sn_{0.4}I_3$, (b) G-$CsPb_{0.6}Sn_{0.4}I_3$, and (c) G-S-$CsPb_{0.6}Sn_{0.4}I_3$ (d) Absorbance (at 600 nm) variation as a function of time duration.

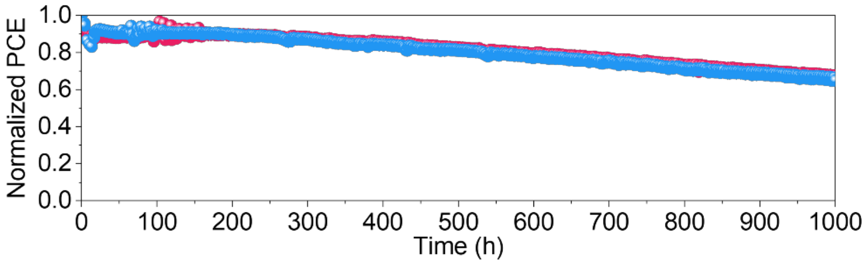

Supplementary Figure 14. Normalized PCE of two other G-S-$CsPb_{0.6}Sn_{0.4}I_3$-based PSCs (unencapsulated) as a function of time upon continuous operation under one-sun intensity illumination (nitrogen atmosphere, ~45 °C). The stabilities are close to that in Figure 6c, confirming reproducibility.

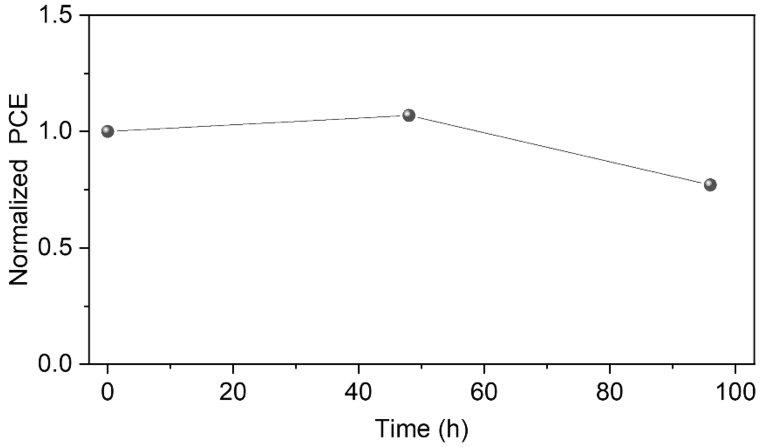

Supplementary Figure 15. Stability of a G-S-$CsPb_{0.6}Sn_{0.4}I_3$-based PSC after storage in a nitrogen-filled glovebox at 85 ºC.

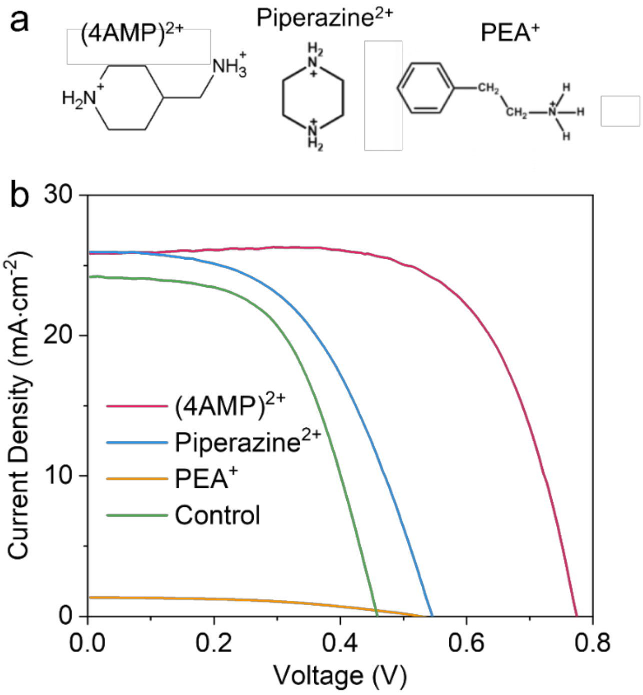

c

	V_{oc} (V)	J_{sc} (mA.cm^{-2})	FF	PCE (%)
Control	0.459	24.159	0.563	6.235
$(4AMP)^{2+}$	0.774	25.87	0.678	13.366
Piperazine^{2+}	0.545	25.94	0.513	7.255
PEA^{+}	0.478	1.35	0.451	0.291

Supplementary Figure 16. (a) Molecular structures of $(4AMP)^{2+}$, piperazine^{2+}, and PEA^{+} cations. (b) *J-V* curves of the PSC devices made with G-$CsPb_{0.6}Sn_{0.4}I_3$ thin films that are then surface-treated with $(4AMP)I_2$, piperazine iodide, and PEAI. (c) Table summarizing the *J-V* parameters extracted from (b).

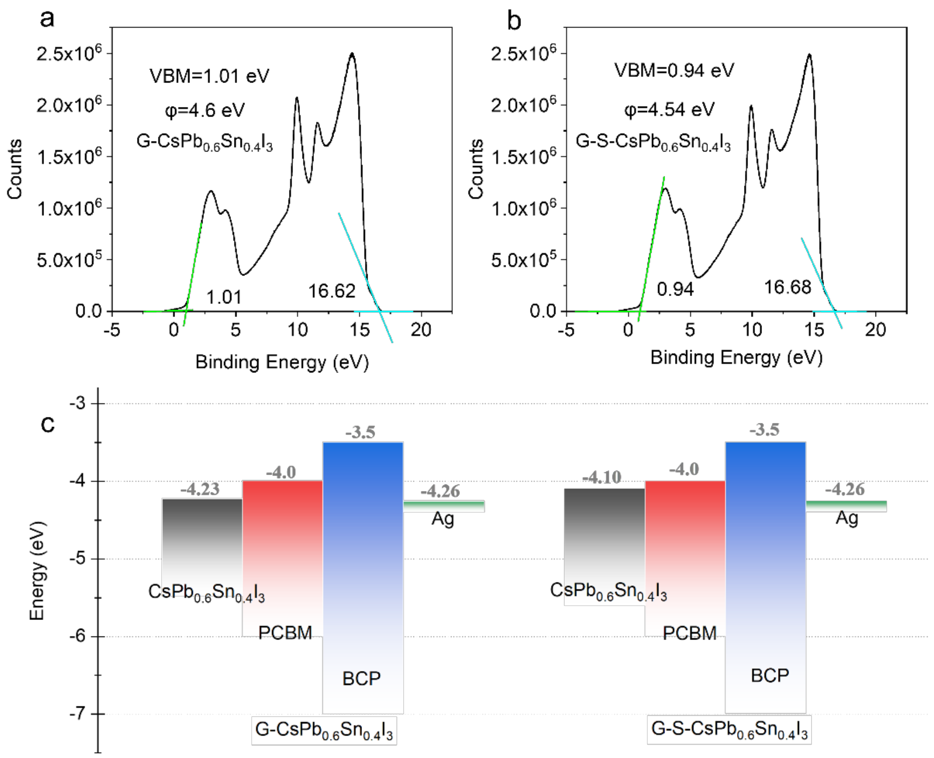

Supplementary Figure 17. UPS spectra of: (a) G-$CsPb_{0.6}Sn_{0.4}I_3$ and (b) G-S-$CsPb_{0.6}Sn_{0.4}I_3$ thin films. (c) Comparison of energy-level alignments for PSCs based on G-$CsPb_{0.6}Sn_{0.4}I_3$ and G-S-$CsPb_{0.6}Sn_{0.4}I_3$ thin films. The conduction band maximum is obtained using the UPS spectra.

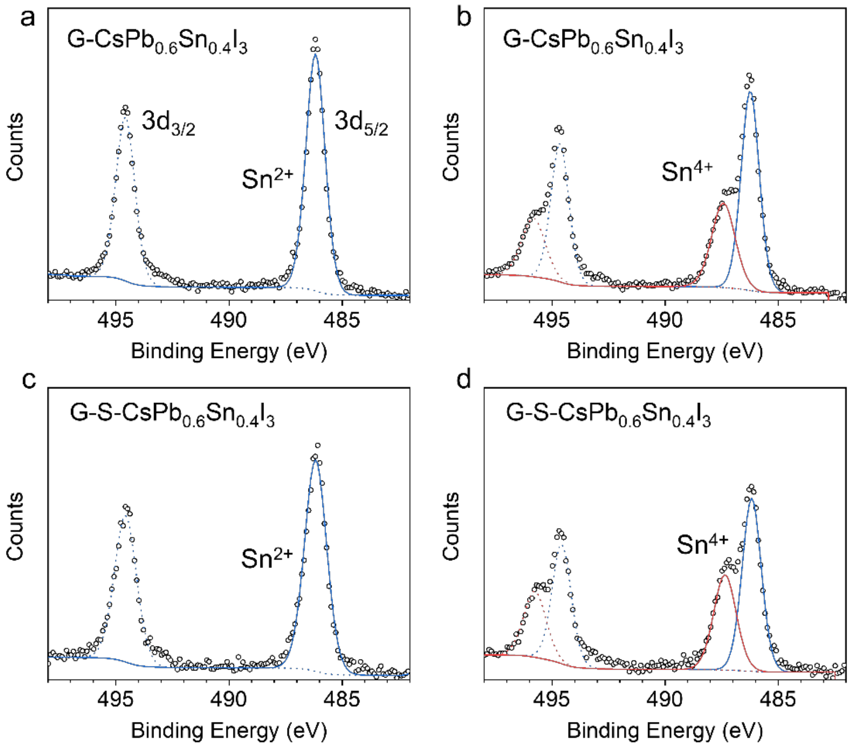

Supplementary Figure 18. XPS spectra measured on the G-$CsPb_{0.6}Sn_{0.4}I_3$ thin film: (a) before and (b) after 1.5-h exposure to ambient air. XPS spectra measured on the G-S-$CsPb_{0.6}Sn_{0.4}I_3$ thin film: (c) before and (d) after 1.5-h exposure to ambient air.

Supplementary Table 1. TA spectra fitting parameters of the bi-exponential functions with two time-constants τ_1 and τ_2.

Sample	A_1	τ_1 (ps)	A_2	τ_2 (ps)	τ_{avg} (ps)
N-$CsPb_{0.6}Sn_{0.4}I_3$	2.54×10^{-3}	13.21	3.01×10^{-3}	106.74	97.89
G-$CsPb_{0.6}Sn_{0.4}I_3$	2.58×10^{-3}	15.36	5.76×10^{-3}	153.92	148.00
G-S-$CsPb_{0.6}Sn_{0.4}I_3$	3.29×10^{-3}	37.46	3.95×10^{-3}	291.73	267.16

Supplementary Table 2. Summary of device stability results of low-bandgap PSCs in this work and in the literature.

Perovskite composition	Stabilization method	Device stability	Ref.
$CsPb_{0.6}Sn_{0.4}I_3$	Inorganic perovskite composition and interface functionalization	80% retention of the initial efficiency after 653 h under continuous one-sun illumination (unencapsulated device; continuous flow of nitrogen gas; operational stability);<br>70% retention of the initial efficiency after 1045 h under continuous one-sun illumination (unencapsulated device; continuous flow of nitrogen gas; operational stability);<br>100% retention of the initial efficiency after 2800 h in the nitrogen-filled glove box (unencapsulated device, shelf stability);<br>20% enhancement of the initial efficiency after 1000 h (periodical exposure to the ambient atmosphere (RT, <20% RH) for 2~3 h every 3~4 days, unencapsulated device, shelf stability);<br>77% retention of the initial efficiency after 100 h in a nitrogen-filled glove box at 85 °C (unencapsulated device, shelf stability)	This work
$(FASnI_3)_{0.6}(MAPbI_3)_{0.4}$	Grain-boundary passivation GuaSCN additive	80% retention of the initial efficiency after ~200 h under continuous one-sun illumination (encapsulated device; operational stability)	1
$(FAPbI_3)_{0.7}(CsSnI_3)_{0.3}$	Grain-boundary functionalization using $SnF_2{\cdot}3FACl$ additive	98.3% retention of the initial efficiency after 288 h in the nitrogen-filled glove box (shelf stability);<br>90% retention of its initial efficiency after 288 h in ambient air (20% RH) (shelf stability)	2
$(FASnI_3)_{0.6}(MAPbI_3)_{0.4}$	Cl-based additive	85% retention of the initial efficiency after ~80 h under continuous one-sun illumination (encapsulated device; operational stability);<br>94% retention of the initial efficiency after 45 days in the ambient air (shelf stability)	3
$MA_{0.5}FA_{0.5}Pb_{0.75}Sn_{0.25}I_3$	Thin film optimization plus charge-transport layer engineering	80% retention of the initial efficiency for 12 days in the ambient air (30–40% RH) (shelf stability);<br>94% retention of its initial efficiency after 30 days shelf stability in the nitrogen atmosphere (shelf stability)	4
$(t\text{-}BA)_2(FA_{0.85}Cs_{0.15})_{n-1}(Pb_{0.6}Sn_{0.4})_nI_{3n+1}$	2D/3D composite	47% retention of the initial efficiency after 2000 h in the nitrogen-filled glove box (shelf stability);	5
$FASn_{0.5}Pb_{0.5}I_3$	Sn(II)-based additive	85% retention of the initial efficiency after 100 h in the nitrogen-filled glove box (under LED	6

		illumination) (shelf stability);	
$MAPb_{0.5}Sn_{0.5}I_3$	Incorporation of fluoroalkyl-substituted fullerene	50% retention of the initial efficiency after 15 days in the ambient air (60±5% RH) (shelf stability);<br>95% retention of the initial efficiency after 15 days in the nitrogen-filled glove box (shelf stability)	7
$MAPb_{0.75}Sn_{0.25}I_3$	C60 additive	80% retention of the initial efficiency after 7 days in the ambient air (30 to 50% RH) (shelf stability)	8
$MASn_{0.5}Pb_{0.5}I_3$	Cs^+ incorporation	76% retention of the initial efficiency after 20 days in the ambient air (35 ± 5% RH) (shelf stability)	9
$FAPb_{0.7}Sn_{0.3}I_3$	MASCN additive	98% retention of the initial efficiency after 30 days in the nitrogen-filled glove box (shelf stability)	10
$FAPb_{0.75}Sn_{0.25}I_3$	Composition and Interface Engineering	92% retention of the initial efficiency after 46 days in the nitrogen atmosphere (shelf stability)	11
$FA_{0.75}Cs_{0.25}Sn_{0.5}$Pb0.5$I_3$	MACl vapor treatment	100% retention of the initial efficiency after 30 h under continuous one-sun illumination in a nitrogen environment (operational stability)	12
$FA_{0.7}MA_{0.3}Sn_{0.3}Pb_{0.7}I_3$	Two-step processing method	80% retention of the initial efficiency after 30 days in the nitrogen-filled glove box (shelf stability)	13
$MAPb_{0.5}Sn_{0.5}(I_{0.8}Br_{0.2})_3$	Br additive	92% retention of the initial efficiency after 30 days in the glove box (shelf stability)<br>44% retention of the initial efficiency after 14 days in the ambient air (30-50% RH) (shelf stability)	14
$MA_{0.5}FA_{0.5}Pb_{0.5}Sn_{0.5}I_3$	Ascorbic acid additive	99% retention of the initial efficiency after 30 days in the nitrogen-filled glove box (shelf stability)	15
$MASn_{0.75}Pb_{0.25}I_{3-x}Cl_x$	$SnCl_2$, SnF_2 additive	98% retention of the initial efficiency after 4000 hours in the nitrogen-filled glove box (shelf stability)	16

Supplementary References:

1. Tong, J., *et al.* Carrier lifetimes of >1 μs in Sn-Pb perovskites enable efficient all-perovskite tandem solar cells. *Science* **364**, 475-479 (2019).
2. Zong, Y., Zhou, Z., Chen, M., Padture, N. P., Zhou, Y. Lewis-adduct mediated grain-boundary functionalization for efficient ideal-bandgap perovskite solar cells with superior stability. *Adv. Energy Mater.* **8**, 1800997 (2018).
3. Zhao, D., *et al.* Efficient two-terminal all-perovskite tandem solar cells enabled by high-quality low-bandgap absorber layers. *Nat. Energy* **3**, 1093-1100 (2018).
4. Yang, Z., *et al.* Stable low-bandgap Pb-Sn binary perovskites for tandem solar cells. *Adv. Mater.* **28**, 8990-8997 (2016).
5. Ramirez, D., *et al.* Layered mixed tin–lead hybrid perovskite solar cells with high stability. *ACS Energy Lett.* **3**, 2246-2251 (2018).

6. Liu, J., *et al.* $FAPb_{1-x}Sn_xI_3$ mixed metal halide perovskites with improved light harvesting and stability for efficient planar heterojunction solar cells. *J. Mater. Chem. A* **5**, 9097-9106 (2017).
7. Rajagopal, A., Liang, P.-W., Chueh, C.-C., Yang, Z., Jen, A. K. Y. Defect passivation via a graded fullerene heterojunction in low-bandgap Pb–Sn binary perovskite photovoltaics. *ACS Energy Lett.* **2**, 2531-2539 (2017).
8. Liu, C., Li, W., Li, H., Zhang, C., Fan, J., Mai, Y. C60 additive-assisted crystallization in $CH_3NH_3Pb_{0.75}Sn_{0.25}I_3$ perovskite solar cells with high stability and efficiency. *Nanoscale* **9**, 13967-13975 (2017).
9. Liu, X., *et al.* Improved efficiency and stability of Pb–Sn binary perovskite solar cells by Cs substitution. *J. Mater. Chem. A* **4**, 17939-17945 (2016).
10. Lian, X., *et al.* Highly efficient Sn/Pb binary perovskite solar cell via precursor engineering: a two-step fabrication process. *Adv. Funct. Mater.* **29**, 1807024 (2019).
11. Chi, D., *et al.* Composition and interface engineering for efficient and thermally stable Pb-Sn mixed low-bandgap perovskite solar cells. *Adv. Funct. Mater.* **28**, 1804603 (2018).
12. Leijtens, T., *et al.* Tin–lead halide perovskites with improved thermal and air stability for efficient all-perovskite tandem solar cells. *Sustainable Energy Fuels* **2**, 2450-2459 (2018).
13. Wang, Y., Fu, W., Yan, J., Chen, J., Yang, W., Chen, H. Low-bandgap mixed tin–lead iodide perovskite with large grains for high performance solar cells. *J. Mater. Chem. A* **6**, 13090-13095 (2018).
14. Yang, Z., Rajagopal, A., Jen, A. K. Ideal bandgap organic-inorganic hybrid perovskite solar cells. *Adv. Mater.* **29**, 1704418 (2017).
15. Xu, X., *et al.* Ascorbic acid as an effective antioxidant additive to enhance the efficiency and stability of Pb/Sn-based binary perovskite solar cells. *Nano Energy* **34**, 392-398 (2017).
16. Tsai, C.-M., *et al.* Role of tin chloride in tin-rich mixed-halide perovskites applied as mesoscopic solar cells with a carbon counter electrode. *ACS Energy Lett.* **1**, 1086-1093 (2016).
