# Supplementary material for: Sub-1.4eV bandgap inorganic perovskite solar cells with long-term stability
Source: Nat Commun. 2020 Jan 9;11:151. doi: 10.1038/s41467-019-13908-6 (PMC6952449; doi:10.1038/s41467-019-13908-6)
Supplement: Supplementary file 2 — Reporting Summary [file 41467_2019_13908_MOESM2_ESM.pdf]

nature research

Corresponding author(s): Jing Feng; Nitin P. Padture; Yuanyuan Zhou

Solar Cells Reporting Summary

Nature Research wishes to improve the reproducibility of the work that we publish. This form is intended for publication with all accepted papers reporting the characterization of photovoltaic devices and provides structure for consistency and transparency in reporting. Some list items might not apply to an individual manuscript, but all fields must be completed for clarity.

For further information on Nature Research policies, including our data availability policy, see Authors & Referees.

Experimental design

Please check: are the following details reported in the manuscript?

1. Dimensions

Item	Yes	No	Details
Area of the tested solar cells	[x]	[ ]	Methods - Solar cell performance testing
Method used to determine the device area	[x]	[ ]	Methods - Solar cell performance testing

2. Current-voltage characterization

Item	Yes	No	Details
Current density-voltage (J-V) plots in both forward and backward direction	[x]	[ ]	Figure 5d
Voltage scan conditions *For instance: scan direction, speed, dwell times*	[x]	[ ]	Methods - Solar cell performance testing
Test environment *For instance: characterization temperature, in air or in glove box*	[x]	[ ]	Methods - Solar cell performance testing
Protocol for preconditioning of the device before its characterization	[x]	[ ]	Methods - Solar cell performance testing
Stability of the J-V characteristic *Verified with time evolution of the maximum power point or with the photocurrent at maximum power point; see ref. 7 for details.*	[x]	[ ]	Methods - Long-term stability testing

3. Hysteresis or any other unusual behaviour

Item	Yes	No	Details
Description of the unusual behaviour observed during the characterization	[ ]	[x]	Typical hysteresis
Related experimental data	[x]	[ ]	Figure 5d

4. Efficiency

Item	Yes	No	Details
External quantum efficiency (EQE) or incident photons to current efficiency (IPCE)	[ ]	[x]	Unavailability of working lab facilities at the time of experiments
A comparison between the integrated response under the standard reference spectrum and the response measure under the simulator	[ ]	[x]	No EQE or IPCE data
For tandem solar cells, the bias illumination and bias voltage used for each subcell	[ ]	[x]	Not tandem solar cells

5. Calibration

Item	Yes	No	Details
Light source and reference cell or sensor used for the characterization	[x]	[ ]	Methods - Solar cell performance testing
Confirmation that the reference cell was calibrated and certified	[x]	[ ]	Methods - Solar cell performance testing

Calculation of spectral mismatch between the reference cell and the devices under test

☐ Yes
☒ No

The reference cell is silicon solar cell and its light absorption range is larger than the devices in the manuscript.

6. Mask/aperture

Size of the mask/aperture used during testing

☒ Yes
☐ No

Methods - Solar cell performance testing

Variation of the measured short-circuit current density with the mask/aperture area

☐ Yes
☒ No

We don't have mask with different area, we will do this in the future study.

7. Performance certification

Identity of the independent certification laboratory that confirmed the photovoltaic performance

☐ Yes
☒ No

Device efficiency still needs to be improved.

A copy of any certificate(s)
Provide in Supplementary Information

☐ Yes
☒ No

No certification

8. Statistics

Number of solar cells tested

☒ Yes
☐ No

Caption in Figure 5c

Statistical analysis of the device performance

☒ Yes
☐ No

Figure 5c

9. Long-term stability analysis

Type of analysis, bias conditions and environmental conditions
For instance: illumination type, temperature, atmosphere humidity, encapsulation method, preconditioning temperature

☒ Yes
☐ No

Methods - Long-term stability testing
